# Investigation of the Optimal Prime Boost Spacing Regimen for a Cancer Therapeutic Vaccine Targeting Human Papillomavirus

**DOI:** 10.3390/cancers14174339

**Published:** 2022-09-05

**Authors:** Diane M. Da Silva, Emma A. Martinez, Lies Bogaert, W. Martin Kast

**Affiliations:** 1 Norris Comprehensive Cancer Center, University of Southern California, Los Angeles, CA 90033, USA; 2 Department of Molecular Microbiology & Immunology, University of Southern California, Los Angeles, CA 90033, USA; 3 Department of Obstetrics & Gynecology, University of Southern California, Los Angeles, CA 90033, USA

**Keywords:** prime-boost immunization, tumor immunity, T cell memory, cytotoxic T cells, therapeutic vaccine

## Abstract

**Simple Summary:**

The selection of a therapeutic vaccine schedule can influence the magnitude, efficacy, and durability of immune responses. This study aims to test different prime-boost intervals using a model vaccine in a well-established tumor model system to investigate how the timing of repetitive antigen exposure impacts the induction of effector and memory T cells. Identifying the vaccine schedule most likely to induce durable protective anti-tumor immunity will facilitate decisions made to balance the induction of highly cytotoxic effector T cells and the generation of long-term immunologic memory.

**Abstract:**

Therapeutic vaccine studies should be designed to elicit durable, high magnitude, and efficacious T cell responses, all of which can be impacted by the choice of the vaccination schedule. Here, we compare different prime-boost intervals (PBI) in a human papillomavirus (HPV) model using a HPV16E7E6 Venezuelan equine encephalitis virus replicon particle (VRP) vaccination to address the optimal boosting schedule, quality of immune response, and overall in vivo efficacy. Six different vaccine regimens were tested with each group receiving booster vaccinations at different time intervals. Analysis of T-cell responses demonstrated a significant HPV16 E7 specific CD8^+^ T cell response with at minimum a one-week PBI between antigen re-exposure. Significant E7-specific in vivo cytotoxicity was also observed with longer PBIs. Additionally, longer PBIs led to an enhanced memory recall response to tumor challenge, which correlated with differential expansion of T cell memory subsets. Our findings imply that when using alphavirus vector platforms as a vaccination strategy, a one-week PBI is sufficient to induce high magnitude effector T cells with potent anti-tumor activity. However, longer PBIs lead to enhanced long-term protective anti-tumor immunity. These findings have implications for therapeutic vaccine clinical trials in which shorter intervals of prime-boost regimens may lead to suboptimal durable immune responses.

## 1. Introduction

Therapeutic vaccines aim to stimulate cellular immune responses to eliminate transformed or virally infected cells. They target antigens presented by cancer cells and aim to control malignancies by activating the patient’s own cellular immune response to recognize and kill cancer cells that express tumor-specific antigens. Effector T cells (T_EFF_) have a potent anti-tumor activity, but their effect is short-term. In contrast to effector cells, memory cells provide more robust and enduring protection against tumors [1,2,3]. Several different subsets of memory cells have been recognized that are associated with vaccine efficacy. Central memory cells (T_CM_) are cells that are home to lymph nodes through the expression of chemokine receptor 7 (CCR7) and CD62L. They quickly proliferate and differentiate into effector cells upon secondary stimulation. In contrast, effector memory cells (T_EM_) migrate to peripheral tissues and show a limited proliferative capacity but rapidly produce effector cytokines in response to antigen stimulation. Both subsets are long-lived and offer broad protection [2,4]. 

Therapeutic vaccines should be designed with an emphasis on efficacy, durability, magnitude, and breadth [5]. However, surprisingly little information is available about determining the optimal boosting interval for various vaccine platforms. Standards frequently lack when prime-boost intervals (PBI) in vaccination studies need to be determined. PBIs are often set at several weeks to optimize the generation of memory T cells, which need at least 40 days for full differentiation [6,7]. Boosting too early may lead to suboptimal T cell responses or terminal differentiation [8,9]. However, when vaccination needs to be applied in a therapeutic setting, especially when treating fast-growing tumors, a long PBI may not be able to keep in pace with the tumor growth. Therefore, a shorter interval between subsequent vaccinations often needs to be applied in therapeutic settings, although no evidence exists that this strategy will yield sufficient CD8^+^ cytotoxic T cells. Additionally, many prophylactic studies use long intervals, whereas shorter intervals that accelerate the generation of new data could reduce costs and might be as efficient. 

Cervical cancer is the fourth most prevalent cancer among women, accounting for over 570,000 new cases per year worldwide [10,11]. High-risk types of human papillomavirus (HPV) are a necessary, albeit non-sufficient, cause for cervical cancer development [12]. The causal association between genital HPV infection and cervical cancer has prompted substantial interest in the development of prophylactic and therapeutic vaccines against high-risk HPV types. The most frequently targeted antigens in therapeutic settings are the E6 and E7 proteins because they are oncogenic and sustained expression is required for the maintenance of the cancerous phenotype [13,14]. Existing murine HPV tumor models permit pre-clinical evaluation of various vaccine platforms, immune modulators, or combinations thereof in order to assess immunogenicity and efficacy, and establish early proof of concept [15,16,17].

With the goal of generating evidence-based guidance for the optimal use of a particular vaccine platform for immunotherapeutic development, the aim of our study was to compare and assess different homologous prime-boost intervals using a therapeutic vaccine model comprised of an HPV16 E6 and E7-expressing replication incompetent Venezuelan equine encephalitis virus replicon particles (VRP) [18]. We evaluated the number and phenotype of generated antigen-specific T cells, their cytotoxic capacity, and protection against tumor challenges in both a prophylactic and therapeutic vaccine setting. 

## 2. Materials and Methods

### 2.1. Mice and Cell Lines

Pathogen-free female C57BL/6 mice, from 6 to 8 weeks old, were purchased from Taconic Farms. Tumor challenge studies were performed using the C3.43 cell line, an in vivo passaged derivative of the C3 HPV16 transformed murine tumor cell line [15,19]. C3.43 cells were cultured in an Iscove’s modified Dulbecco’s medium supplemented with 10% fetal bovine serum. All animal studies were in compliance and approved by the University of Southern California Institutional Animal Care and Use Committee.

### 2.2. Vaccine Regimens

**Figure 1:**
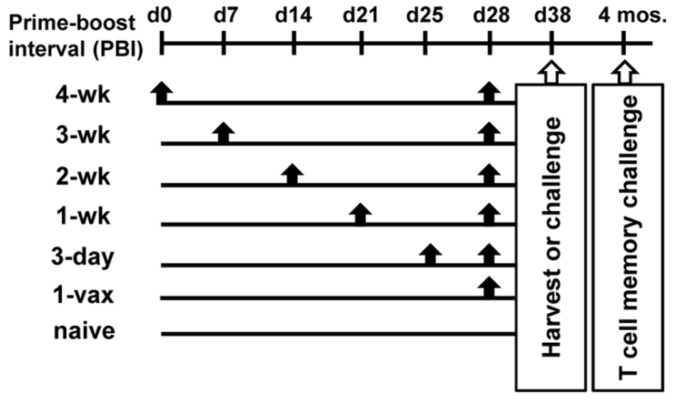
Schematic of prime-boost interval schedule. Six different vaccine regimens were used in this study: in each experiment five groups of mice (*n* = 5–10/group) received a prime vaccination with HPV16 VRP, followed by boosting with the same vaccine after different intervals (4 weeks, 3 weeks, 2 weeks, 1 week, and 3 days, denoted by black arrows). One group received only one vaccination without boosting (1-vax). One group did not receive any vaccinations (naïve mice). Vaccination schedules were planned so that all mice received their final vaccination on the same day. In vitro assays were performed on splenocytes isolated ten days after final injection, or mice were challenged with C3.43 tumors to assess effector T cell response. To analyze memory T cell recall responses, mice were challenged 4 months after the last vaccination without any intervening treatment.

HPV16E7E6 TetM replication incompetent Venezuelan equine encephalitis virus replicon particles (VRP) were produced as previously described [20], with the exception that the VRP used in this study were produced in Vero cells as a GMP-grade quality batch for mouse and non-human primate studies. In vivo titration of the GMP-grade VRP was performed; an effective dose of 1 × 10^7^ infectious units (IU) per immunization was used throughout this study. Mice were vaccinated with 10^7^ IU of HPV16E7E6 VRP intramuscularly (i.m.) in each quadriceps in 20 μL phosphate buffered saline (PBS) at indicated intervals. Vaccination schedules were planned so that all mice received their final vaccination on the same day. The timeline of the performed studies is graphically represented in Figure 1.

### 2.3. Peptides

The H2-Db-binding peptide, HPV16 E7_(49–57)_ RAHYNIVTF peptide [15], and the PSCA_(23–31)_ AQMNNRDCL peptide from prostate stem cell antigen [21], were synthesized at the University of Chicago (Chicago, IL, USA) and purified by reverse phase high-performance liquid chromatography (HPLC). Purity was assessed by analytic HPLC and determined to be >95% pure.

### 2.4. Enzyme-Linked Immunospot Assay (ELISpot)

Functional IFNγ producing tumor antigen–specific cells specific for HPV16 E7_(49–57)_ were detected 10 days after final vaccination. A total of 5 × 10^6^ freshly isolated splenocytes (*n* = 5 per group, 3 independent experiments) were stimulated without or with E7 peptide (final concentration 1 µg/mL) and with 5 IU interleukin (IL)-2/mL in culture medium for 24 h. Multiscreen HA plates (EMD Millipore, Burlington, MA, USA.) were coated with 10 μg/mL anti-IFNγ antibody (BD Biosciences, San Jose, CA, USA). Plates were washed and blocked with culture medium. Splenocytes were added to the coated plates in 2-fold serial dilutions ranging from 5 × 10^5^ to 6.25 × 10^4^ cells per well. After 20 h, plates were washed and incubated with 1 μg/mL of biotinylated anti-IFNγ antibody (BD Biosciences). Washed plates were incubated with 100 μL of 1:4000 diluted streptavidin–horseradish peroxidase (Sigma Aldrich, St. Louis, MO, USA) per well. Spots were developed using 3-amino-9-ethylcarbazole (Sigma, St. Louis, MO, USA) for 5 min, and the reaction was stopped with water. Spots were counted using the Zeiss KS enzyme-linked immunospot system. Assays were performed in triplicate, and results were calculated as spot-forming cells (SFC) per 10^6^ splenocytes after subtracting medium background. 

### 2.5. MHC Tetramer Analysis

To enumerate and phenotype HPV16 E7_(49–57)_ specific CD8^+^ T cells, splenocytes were stained with H-2Db tetramers containing the HPV16 E7_(49–57)_ peptide obtained from the National Institute of Allergy and Infectious Diseases Tetramer Facility (Atlanta, GA, USA) at 0.5 μg/mL together with, anti-CD3, and anti-CD8 antibody (BD Biosciences). Gating was done on CD3^+^CD8^+^ lymphocytes, and the percentage of CD8^+^tetramer^+^ T cells was determined (Supplemental Figure S1). In one experiment splenocytes were also stained with antibodies for memory subset phenotyping, (i.e., CD44, CD127, and CD62L; all BD Biosciences, San Jose, CA). Gating was done on CD8^+^/Tet^+^ T cells, and percentages of T_EM_ (CD62L^−^CD127^+^), T_CM_ (CD62L^+^CD127^+^), and T_EFF_ (CD62L^−^CD127^−^) were calculated. All tetramer-positive cells were identified as CD44+ (Supplemental Figure S2). At least 100,000 events were acquired on the Beckman Coulter FC500 flow cytometer and analyzed using CXP software. 

### 2.6. In Vivo Cytoxicity Assay

Naive splenocytes were incubated with either a relevant HPV16E7_(49–57)_ peptide or irrelevant PSCA_(23–31)_ peptide at a concentration of 0.5 µg/mL. Cells with relevant peptide were labeled with 10 mM CFSE using Vybrant CFDA SE Cell Tracer Kit (Life Technologies, Carlsbad, CA, USA) and cells with irrelevant peptide with 0.66 mM CFSE. Cells were mixed in a ratio of 1:1. Ten million CFSE-labeled cells were injected intravenously (i.v.) into vaccinated and control mice (*n* = 5 per group, 10 days after final vaccination). The following day, spleens were harvested and 5 × 10^6^ cells were analyzed on the Beckman Coulter FC500 flow cytometer. At least 5000 CFSE^+^ events were collected. Percentage lysis was calculated as follows: [1 − (% CFSE^hi^ population/% CFSE^low^ population)] × 100.

### 2.7. In Vivo Tumor Studies

In prophylactic studies examining the effect of vaccination with HPV16 VRP, groups of ten 8-week-old female C57BL/6 mice were vaccinated with 10^7^ IU of VRP. Ten days after the last vaccination, mice were challenged subcutaneously (s.c.) in the right flank with 5 × 10^5^ C3.43 tumor cells in 100 µL PBS. Tumor growth was monitored twice weekly with manual calipers in three dimensions. To test long term memory T cell recall post vaccination, mice were vaccinated as described above and then left untouched for 4 months prior to tumor challenge. In a therapeutic setting, mice were challenged with 5 × 10^5^ C3.43 at day 0; 5 days post tumor challenge mice were immunized i.m. with VRP at increasing PBIs. Tumor growth was monitored for 70 days post tumor challenge. Mice were euthanized per University of Southern California Animal Care and Use Committee guidelines when tumor volume exceeded 1500 mm^3^. 

### 2.8. Statistical Analysis

ELISpot, flow cytometry and in vivo cytotoxicity data were analyzed by one-way ANOVA testing, followed by Tukey’s multiple comparisons test for individual group comparisons. Survival was analyzed by the log-rank (Mantel-Cox) test. All statistical analyses were performed using the GraphPad Prism version 9.3.1 software (GraphPad Software, San Diego, CA, USA).

## 3. Results

### 3.1. Effect of Increasing Prime-Boost Intervals on Magnitude of Induced Effector HPV Specific CD8^+^ T Cell Population

**Figure 2:**
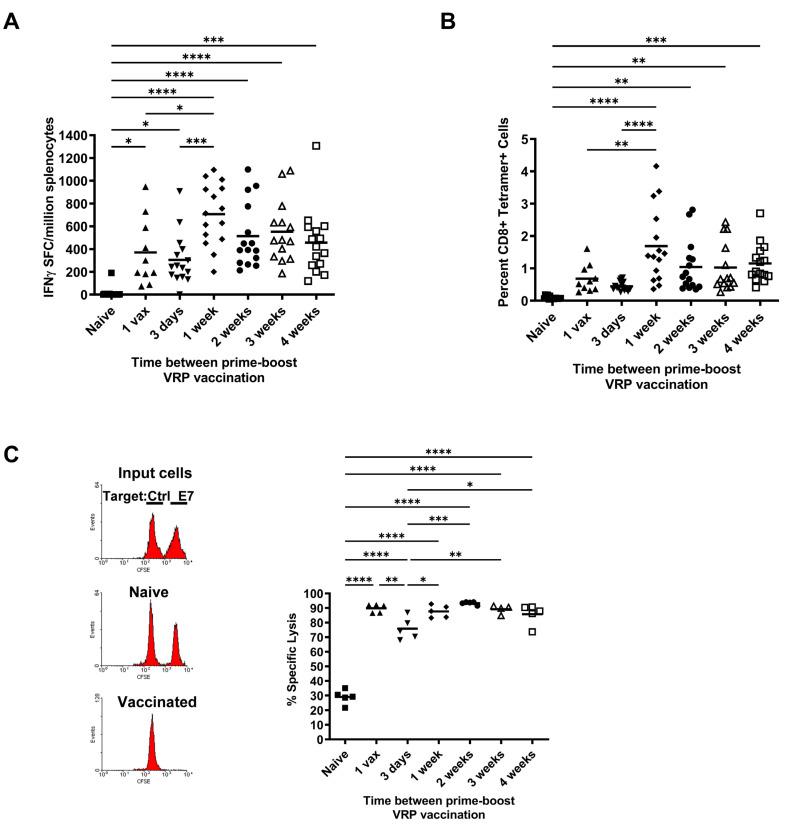
Ex vivo analysis of T-cell response after different prime-boost intervals. C57BL/6 mice (*n* = 5/group per experiment) were immunized i.m. with 1 × 10^7^ IU HPV16 E7E6 VRP according to the vaccination schedule in Figure 1. (**A**) HPV16 E7-specific IFNγ secretion by ELISpot assay. Shown are the number of spot-forming cells (SFC) per million splenocytes for each mouse from three independent experiments with the mean indicated by the horizontal bar. Mean SFC from all vaccinated mice were significantly increased compared to naïve mice (range of *p* < 0.05 to *p* < 0.0001, one-way ANOVA). (**B**) Splenocytes were tested for binding of H2-D^b^ MHC tetramers loaded with HPV16 E7_(49–57)_ peptide. Shown is the percentage of E7 tetramer-binding CD8^+^ T cells for each group of mice from three independent experiments with the mean indicated by the horizontal bar. Mice vaccinated at 4 wk, 3 wk, 2 wk, and 1 wk prime-boost exhibited a greater mean number of E7 tetramer positive CD8^+^ T cells compared to naïve mice (range of *p* < 0.01 to *p* < 0.0001). (**C**) In vivo cytotoxicity assay. Naïve C57Bl/6 splenocytes were loaded with E7_(49–57)_ peptide or irrelevant control D^b^-binding peptide, then labeled with a high dose (E7peptide-loaded) or low dose (control peptide-loaded) of CFSE. Shown are representative histogram plots of input labeled cell populations and CFSE populations in a naïve or vaccinated mouse. Calculated E7-specific cytotoxicity is shown for all groups. Specific lysis in all vaccinated groups is significantly different from naïve mice (*p* < 0.0001). * *p* < 0.05, ** *p* < 0.01, *** *p* < 0.001, **** *p* < 0.0001 (One-way ANOVA, Tukey’s post-test). All significant pairwise comparisons are indicated in the figure. All other pairwise comparisons were not significant.

We previously have reported that replication incompetent VEE replicon particles (VRP) expressing HPV16 *E6* and *E7* mutated genes are highly immunogenic and induce CD8^+^ T cells that exhibit potent anti-tumor efficacy in several HPV-induced murine tumor models [17,18,20]. These studies used varying PBIs ranging from two weeks apart in the prophylactic setting to 5–7 day injection intervals in the therapeutic setting. In these cases, it was not clear which regimen induces the highest magnitude, most durable responses. To thoroughly investigate the role of different PBIs on the induction of antigen-specific CD8^+^ T cells, we immunized groups of mice with HPV16E7E6 VRP as shown schematically in Figure 1 and evaluated the quantity and functionality of the resulting antigen-specific T cells by MHC tetramer and IFNγ ELISpot analysis. HPV16E7E6 VRPs were highly immunogenic in inducing antigen-specific T cells, even after just one vaccination (Figure 2). All vaccinated groups showed significant increases in HPV16 E7–specific T cell responses by IFNγ secretion compared to unvaccinated naïve mice (*p* < 0.05, naïve vs. 1-vax, 3-day; *p* < 0.0001, naïve vs. 1 wk, 2 wk, 3 wk, 4 wk PBIs) (Figure 2A). By tetramer positivity, PBIs of 1 wk, 2 wk, 3 wk, and 4 wk were significant compared to naïve mice (*p* < 0.0001, naïve vs. 1 wk; *p* < 0.01, naïve vs. 2 wk, 3 wk; *p* < 0.001, naïve vs. 4 wk) (Figure 2B). The 1 wk PBI was also significantly higher than the 1 vax (*p* < 0.01) and 3 day PBI (*p* < 0.0001) in mean frequency of tetramer-positive cells (Figure 2B). The highest numbers of HPV16 E7_(49–57)_ peptide-specific T cells were induced with a PBI of one week, as seen both by ELISpot (Figure 2A) and tetramer analysis (Figure 2B). Longer PBIs of 2-wk, 3-wk, or 4-wk did not significantly change the frequency of HPV-specific T cells compared to the 1-wk boost (Figure 2B). Since the E7_(49–57)_ peptide is known to be a subdominant CD8^+^ T cell epitope in C57BL/6 mice [22], and H2-Db-E7_(49–57)_ tetramer binding was only observed on CD8^+^ T cells, these data indicate that a minimum of one week between vaccinations is likely to be optimal for generating a high number of effector antigen-specific CD8^+^ T cells using a viral therapeutic vaccine platform. 

We next determined the cytolytic potential of the induced HPV-specific CD8^+^ cytotoxic T cells (CTLs) generated by the different prime-boost regimens to gauge how likely they are to recognize and kill HPV-expressing tumor cells. In vivo cytotoxicity studies were performed by adoptively transferring naive differentially-labeled CFSE^+^ splenocytes loaded with the relevant HPV16E7_(49–57)_ peptide or a control irrelevant PSCA_(23–31)_ peptide to groups of mice immunized as indicated in Figure 1. Loss of CFSE^hi^ E7-pulsed target cells is indicative of the in vivo effectiveness of the different PBIs in inducing CTLs. Our data demonstrate that all prime-boost regimens, including administration of a single dose, resulted in nearly 100% lysis of HPV16 E7_(49–57)_ loaded target cells compared to naïve mice (*p* < 0.0001) (Figure 2C). Interestingly, when comparing vaccinated groups against each other, a PBI of 3 days resulted in a significantly lower lysis than the other regimens (range, *p* < 0.05 to *p* < 0.001), including a single dose of VRP vaccine, suggesting that a very short interval of exposure to antigen may be detrimental to the formation of the pool of effector CTLs.

### 3.2. Vaccination Protects against Tumor Challenge Independent of Prime-Boost Interval in a Prophylactic HPV16 Tumor Model

Since prime-boost regimens resulted in the expansion of E7-specific T cells with significant cytolytic activity, we next sought to determine whether these T cells were functional in their ability to lyse HPV16-transformed syngeneic tumor cells. To determine if there was a difference in protection against tumor challenge between PBIs, groups of mice received VRP injections as per Figure 1 and were subsequently challenged with C3.43 tumor cells ten days after the final vaccination. Despite lower in vivo cytotoxicity exhibited in the 3-day PBI group against peptide-loaded target cells shown above, all vaccination regimens, including administration of a single dose, resulted in 100% protection against tumor challenge (*p* < 0.0001). None of the vaccinated mice developed any sign of tumor growth in the 60 days following tumor challenge, whereas all naïve mice developed progressively growing tumors resulting in euthanasia (Table 1). These results indicate that in the prophylactic setting, the pool of antigen-specific T cells induced by HPV16 VRP vaccination was functionally capable of recognizing and killing HPV16-expressing tumor cells regardless of PBI, likely due to the high number of E7-specific effector T cells induced even with one dose.

### 3.3. Longer Prime-Boost Intervals Lead to Enhanced Memory Recall Response to Tumor and Differential Induction of Memory T Cell Phenotypes

**Figure 3:**
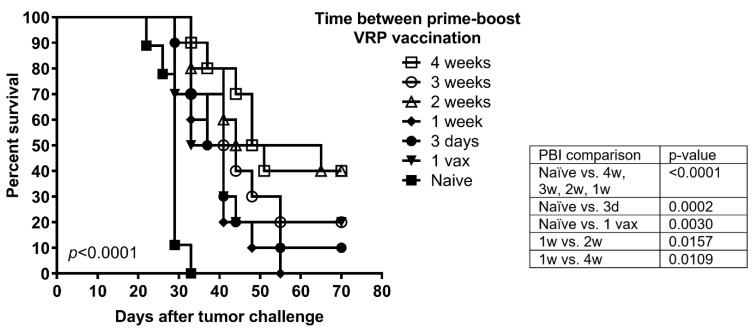
Longer intervals between prime and boost lead to enhanced memory T cell recall responses to tumor several months post vaccination. C57BL/6 mice (*n* = 10/group) were immunized with HPV16 E6E7 VRP according to the vaccination schedule in Figure 1. Four months after the last vaccination, mice were challenged with 5 × 10^5^ C3.43 tumor cells s.c. in the flank to analyze memory recall response induced after varying prime-boost intervals. Median survival of 4 wk PBI was 49.5 days; 3 wk PBI, 42.5 days; 2 wk PBI, 54.5 days; 1 wk PBI, 39 days, 3-day PBI, 39 days; 1 vax, 37 days; naïve, 29 days. All significant pairwise comparisons are indicated in the figure. All other pairwise comparisons were not significant.

To investigate whether increasing PBIs differently affected the pool of memory T cells generated and their memory recall response to tumor, groups of mice were challenged with C3.43 tumor cells four months after the last vaccination, when it is expected that the initial expanded effector T cell population has contracted and only a small percentage of memory T cells remains. Overall survival of the mice irrespective of different boosting schedules was significantly prolonged in comparison to control mice (overall *p* < 0.0001) (Figure 3). With respect to the memory recall anti-tumor response, longer PBIs (4-wk, 3-wk, 2-wk, open symbols) resulted in greater overall survival compared to shorter PBIs (1-wk, 3-day, 1-vax, closed symbols). Indeed, maximum long-term protection was observed with a 4-wk and 2-wk PBI resulting in a significant increase in overall survival compared to a 1-wk PBI (*p* = 0.0109 and *p* = 0.0157, respectively) (Figure 3).

**Figure 4:**
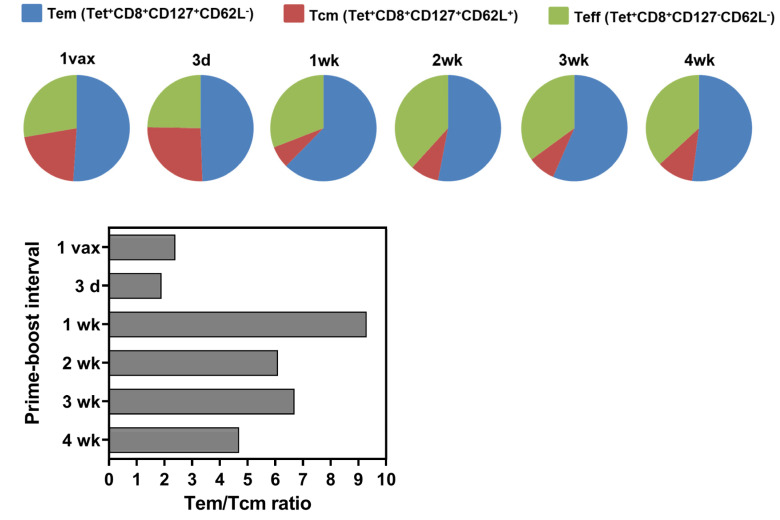
Longer prime-boost interval regimens generate a higher frequency of effector memory T cells. HPV16 E7_(49–57)_ peptide specific T cells identified by MHC tetramer staining were analyzed for T cell effector and memory phenotype. Splenocytes from immunized mice (*n* = 5) were isolated 10 days post boost and stained with E7 tetramer (tet), CD8, CD44, CD127 (IL-7Rα), and CD62L. The percentages of Tem, Tcm, and Teff cell phenotypes were determined after post-analysis Boolean gating on CD3^+^CD8^+^Tet^+^ T cells. Pie charts show the relative frequencies of effector and memory phenotypes within the tetramer positive population. Mice vaccinated at 4 wk, 3 wk, 2 wk, and 1 wk PBIs exhibited similar percentages of each population and greater numbers of T_EM_. The ratios of T_EM_:T_CM_ are shown as bar graphs. T_EM_, effector memory T cell; T_CM_, central memory T cell; Teff, effector T cell.

The overall objective of vaccination is to generate long-lasting memory in order to sustain protective immunity with respect to tumor burden. Hence, we further phenotyped the HPV16E7_(49–57)_ CD8^+^ T cells generated for the proportion of effector memory (T_EM_) and central memory (T_CM_) T cell subsets to investigate whether the PBI affects the expansion of differential subsets of memory T cells. Phenotyping showed an increase in the T_EM_ population relative to T_CM_ in the 1-week PBI, with T_EM_ populations reaching up to 60% of total HPV16 antigen-specific CD8^+^ T cells producing a T_EM_/T_CM_ ratio of 9.3. In contrast, the 3-day PBI resulted in a T_EM_/T_CM_ ratio of 1.9, which was more similar to the single vaccination with a T_EM_/T_CM_ ratio of 2.4 compared to longer PBIs (Figure 4). Thus, longer PBIs resulted in higher frequencies of T_EM_ relative to T_CM_ which may have resulted in providing an improved immediate recall response and protective immunity in response to peripheral subcutaneous tumor challenge long after exposure to HPV antigens during vaccination. Short interval of antigen exposure (3-day PBI) or single dose vaccination resulted in more antigen-specific cells with a T_CM_ phenotype, which may have led to a suboptimal recall response to tumor challenge after four months.

### 3.4. Effect of PBI Regimens on Anti-Tumor Efficacy Using VRP-Based Vaccines in a Therapeutic Tumor Setting

**Figure 5:**
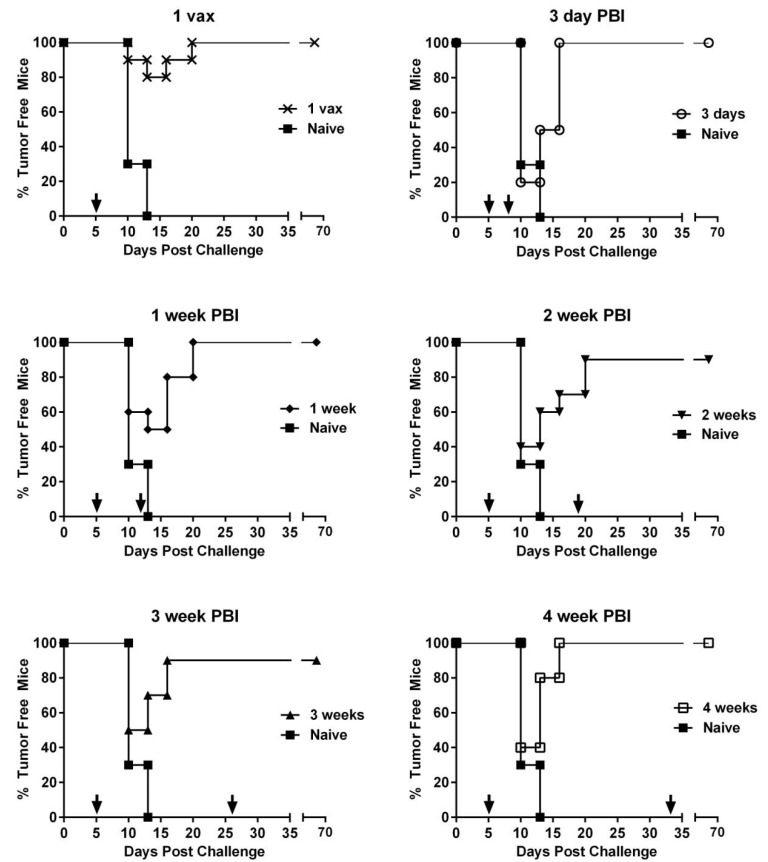
Timing of prime-boost interval does not impact therapeutic efficacy of VRP-based vaccine. C57BL/6 mice (*n* = 10/group) were challenged with 5 × 10^5^ C3.43 tumor cells s.c. in the flank. Mice were immunized i.m. with HPV16 E6E7 VRP at increasing PBIs starting on day 5 post tumor challenge. Vaccinations are indicated by arrows. Each group is graphed individually against naïve, unvaccinated mice. All vaccinated groups were significantly protected compared to naïve group (*p* < 0.0001, log-rank test).

The proliferation rate and aggressiveness of tumor cell growth in vivo have a strong influence on the choice of PBI in a therapeutic cancer setting since the goal of therapeutic vaccines is to tip the balance towards tumor killing rather than T cell exhaustion. To determine the effect of PBIs on the growth of established tumors, we challenged groups of mice with C3.43 tumor cells first and then immunized mice starting 5 days post-challenge with HPV16E7E6 VRP. Tumor growth and tumor clearance were observed for 70 days (Figure 5). Similar to vaccination prior to tumor challenge, a single vaccination with no boosting was sufficient to clear tumors in 100% of mice in comparison to the naive group (*p* < 0.0001). Increasing the boosting intervals up to 4 weeks did not have any significant impact on the tumor growth or clearance of tumor burden. Though not statistically significant, it is interesting to note that in the group of mice boosted at an interval of 3 days, 80% of the mice developed palpable tumors, whereas fewer mice developed palpable tumors in all the other PBI vaccinated groups. Thus, it could be suggested that a very short interval of boosting may not be advantageous in a therapeutic setting, although in this tumor model, all mice eventually were able to resolve their tumors.

## 4. Discussion

Our results indicate that when using a viral vector platform as a therapeutic vaccination strategy, boosting as early as one week is efficient, or even more efficient, than longer PBIs for induction of antigen-specific functional CD8^+^ T cells. The use of a single vaccine dose without additional boosts resulted in a high frequency of functional CD8^+^ T cells by ELISpot, similar to vaccine regimens with booster doses. Both single-dose vaccination and 3-day PBI offered protection in prophylactic tumor challenge experiments but yielded significantly fewer functional cytotoxic CD8^+^ T cells that exhibited T_EM_ characteristics, which might not provide sufficient protection at peripheral sites where tumors might have originated or recurred after treatment. This decrease in functional cytotoxic CD8^+^ T cells was also seen in the 3-day PBI, which we hypothesized to be caused by the activation-induced cell death (ACID) [23]. Therefore, our data suggest that these short interval boosting regimens should be avoided in long-term studies. Although beyond the scope of this study, it would be interesting for future studies to investigate whether these results would be affected by higher tumor challenge doses, differences in the immunodominance of the target antigens, or in the few other HPV-induced tumor models available, including those that involve orthotopic tumor sites. Furthermore, all immunogenicity and tumor efficacy data obtained using mouse models of human disease should be interpreted with caution. Direct translation of mouse data to humans is difficult to predict because of the inherent heterogeneity of individual human tumors, human background genetics and environmental exposures, however, our data clearly indicate that intervals between vaccination can make a difference in the resulting immune response and should be considered in vaccine clinical trial design.

Different types of tumors growing in various anatomical locations and with different antigenic burdens may require different populations of effector and memory T cells for optimal immune protection [1,2,24,25]. A high load of persisting antigen or chronic antigenic stimulation is not ideal for long-term memory but has important benefits on the level of immediate protection by the generation of effector T cells [26]. In viral infection models, it has been shown that long-term protection against infection taking place in lymphoid organs requires T_CM_ cells whereas long-term protection against a peripheral viral challenge requires significant numbers of T_EM_ cells present at the site of viral challenge [27]. At early times after infection, T_EM_ cells dominate the memory pool and provide potent protective immunity, primarily because of their presence at peripheral sites where they can make first contact with the invading pathogen [4]. In therapeutic vaccination settings for peripheral tumors such as cervical cancer, it can be hypothesized that T_EM_ CD8^+^ T cells need to be accessible at distant sites. Phenotyping of antigen-specific CD8^+^ T cells in our study indicates that a minimum PBI of 7 days is required to induce the efficient formation of T_EM_ cells, which have a potent anti-tumor effect in peripheral tissues [1]. Boosting less than 1 week after priming comes too early before the antigen-specific T cells have reached their full proliferative potential after initial stimulation and can result in T cell elimination or exhaustion. Repeated boosting can drive memory cells toward terminal differentiation, which is good if T_EFF_ cells are needed at peripheral tissues. However, it should be kept in mind that antigen overload by successive vaccinations (up to three boosts) may lead to depletion of the T_CM_ cell population [9]. Interestingly, Kaech et. al. [28], found that although the precursors to memory CD8^+^ T cells exist in the effector population 8 days after viral infection, they have not fully acquired the protective qualities of memory cells. This could be explained by differences in memory T cell generation after viral infection versus vaccination. In our study, we found phenotypic differences in memory T cell populations 10 days after antigen exposure via vaccination, however we only assessed T cell function of the resulting memory populations 4 months after antigen exposure.

Based on our studies, initial tumor reduction can be obtained by repetitive 7-day-spaced vaccinations that include one boost, if necessary, combined with other conventional treatment strategies such as chemo- or radiotherapy. Later, when the tumor is under control and no longer life threatening, and especially when recurrences are probable or tumor stem cells have been identified, robust and enduring protection and successful tumor clearance are wanted and T_CM_ cells need to be generated. T_CM_ cells mediate stronger recall responses and will result in better protection in the long term. Although these cells generally are not present at peripheral sites, they become the more potent responders in terms of proliferative potential and in some cases provide more durable immunity [26]. Much longer intervals between boosts are required in order to do so, and reports in the literature suggest that up to 2–3 months between boosting is required to allow effector cells to differentiate into T_CM_ and reset their responsiveness to antigen [6,9,28]. In the cancer setting, a mixture of T _EM_, T _CM,_ and terminal effectors are likely needed for optimal therapeutic efficacy and we suggest this needs to be tested empirically using animal model data to guide human clinical study design rationale.

PBIs may differ depending on the nature of the studied disease and the specific vaccine platform. A study done by Ricupito et al., assessing dendritic cell-based vaccines in both prophylactic as well as therapeutic settings with different prime-boost regimens reported that booster vaccinations were important for the maintenance of Ag-specific CD8^+^ T_CM_ cells in the prophylactic setting, however frequent boosting intervals with up to three booster vaccines hindered cell survival/functionality of T_CM_ cells in the therapeutic setting, suggesting that different boosting schedules affect the outcome of memory T cell differentiation when a tumor was present versus absent [29]. Our findings cannot be compared directly with others as we evaluated a viral vector platform and different PBI regimens in a different tumor model. Different vaccines may exhibit different antigen presentation strategies and other diseases can be localized in different tissues (central versus peripheral versus mucosal) requiring different subsets of T cells under each specific condition. Therefore, it is important and worthwhile for investigators to determine the optimal PBI for their specific platform before starting large clinical trials.

An additional issue worthwhile pointing out is that many therapeutic clinical trials measure antigen-specific immunity as an immunological endpoint. The presence of specific T cells at sites other than the tumor, however, does not necessarily have a positive prognostic value [1]. For example, CD8^+^CD45RA^+^CCR7^−^ effector T cells expressing granzyme B and capable of ex vivo IFNγ production and direct killing of autologous tumor cells were found to make up a significant proportion of circulating tumor-specific cells in the blood of melanoma patients, whether patients had a progressive disease or no evidence of disease following surgical resection of their tumors [30]. Therefore, infiltration of effector T cells into tumor tissue can be a better indicator of a successful anti-tumor response. The absence of clinical efficacy may be related to failure of tumor-specific CTLs being correctly traansported to the tumor site as well as immune-suppressive mechanisms within the tumor microenvironment [31]. We were not able to address this question since all tumors in our study regressed after treatment with even one dose of VRP, therefore our results are limited to peripheral T cell immunophenotyping. Additional studies using human clinical specimens from therapeutic vaccine trials are needed to help clarify these outstanding questions. 

## 5. Conclusions

From the current study we can conclude that functional and effective CD8^+^ anti-tumor immunity can be induced by using a minimum 1-week interval prime-boost regimen with a viral vector. When the study duration or disease state does not allow long prime-boost intervals, boosting can safely be applied 1 week after priming without loss of efficacy. When on the other hand long intervals are required, e.g., to study memory T cell formation and long-term protection, intervals can be prolonged to at least 4 weeks without substantial reduction in CD8^+^ T cell formation. These findings suggest that both short- and long-term immune effector and memory responses to tumor-associated antigens can be influenced by the choice of prime-boost interval in a therapeutic vaccine schedule. Similar informing studies should be performed for other vaccine delivery platforms being tested for cancer therapeutic development to improve vaccine efficacy. 

## Figures and Tables

**Table 1:** Survival of mice receiving different prime-boost regimens ^1^.

Prime-Boost Regimen	Tumor-Free Mice after 60 Days	Significance ^2^
PBI of 4 weeks	10/10	*p* < 0.0001
PBI of 3 weeks	10/10	
PBI of 2 weeks	10/10	
PBI of 1 week	10/10	
PBI of 3 days	10/10	
1 vaccination	10/10	
Naive	0/10	Reference

^1^ Mice challenged with C3.43 tumor cells ten days after last vaccination. ^2^ Log-rank test, unvaccinated mice used as reference group.

## Data Availability

The data presented in this study are available in Supplementary Materials File S1. Further inquiries can be directed to the corresponding author.

## Supplementary Materials

The following supporting information can be downloaded at: https://www.mdpi.com/article/10.3390/cancers14174339/s1, File S1: Source data for all figures and tables. Figure S1. Flow cytometry gating schema for CD8^+^Tetramer^+^ T cell identification. Splenocytes from experimental mice were isolated and stained with antibodies to CD3, CD8, and HPV16 E7(49-57) MHC tetramer reagent. For each sample, a no-tetramer negative control was stained only with CD3 and CD8 antibodies. Lymphocytes were gated on FSC/SSC. Frequency of tetramer positive CD3^+^CD8^+^ T cells was determined after subtraction of the frequency of events in the same gate in the no-tetramer control-stained sample. Shown is a representative example of tetramer positive events in the 1-week PBI group and the naïve group, along with the no-tetramer control stain. Figure S2. Flow cytometry gating schema for CD8^+^Tetramer^+^ memory T cell phenotypic markers. Splenocytes from experimental mice were isolated and stained with antibodies to CD8, CD62L, CD127, CD44 and HPV16 E7(49-57) MHC tetramer reagent. For each sample, a no-tetramer negative control was stained. Lymphocytes were gated on FSC/SSC. Shown are representative examples of tetramer positive events in the 1-week PBI group and the naïve group. (A) Tetramer-positive cells show a phenotype of CD44^+^, indicating antigen-experienced T cells. (B) HPV16 E7 specific T cells identified by tetramer staining show phenotypic markers of central memory (Tcm; CD62L^+^CD127^+^) and effector memory (Tem; CD62L^−^CD127^+^) T cells, in addition to differentiated effector T cells (Teff; CD62L^−^CD127^−^). Samples stained without tetramer were used to define positive tetramer gate position.
