# Supplementary material for: Investigation of the Optimal Prime Boost Spacing Regimen for a Cancer Therapeutic Vaccine Targeting Human Papillomavirus
Source: Cancers (Basel). 2022 Sep 5;14(17):4339. doi: 10.3390/cancers14174339 (PMC9454731; doi:10.3390/cancers14174339)
Supplement: Supplementary file 1 [file cancers-14-04339-s001.zip › Supplemental Figure S1.pdf]

Supplemental Figure S1

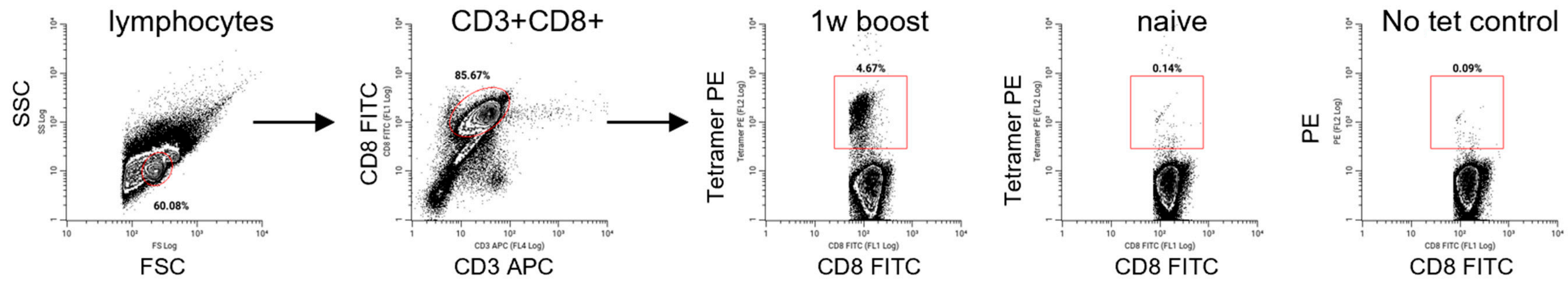

Supplemental Figure S1. Flow cytometry gating schema for CD8+Tetramer+ T cell identification. Splenocytes from experimental mice were isolated and stained with antibodies to CD3, CD8, and HPV16 E7(49-57) MHC tetramer reagent. For each sample, a no-tetramer negative control was stained only with CD3 and CD8 antibodies. Lymphocytes were gated on FSC/SSC. Frequency of tetramer positive CD3+CD8+ T cells was determined after subtraction of the frequency of events in the same gate in the no-tetramer control-stained sample. Shown is a representative example of tetramer positive events in the 1 week PBI group and the naïve group, along with the no-tetramer control stain.
