# Supplementary material for: Investigation of the Optimal Prime Boost Spacing Regimen for a Cancer Therapeutic Vaccine Targeting Human Papillomavirus
Source: Cancers (Basel). 2022 Sep 5;14(17):4339. doi: 10.3390/cancers14174339 (PMC9454731; doi:10.3390/cancers14174339)
Supplement: Supplementary file 1 [file cancers-14-04339-s001.zip › Supplemental Figure S2.pdf]

Supplemental Figure S2

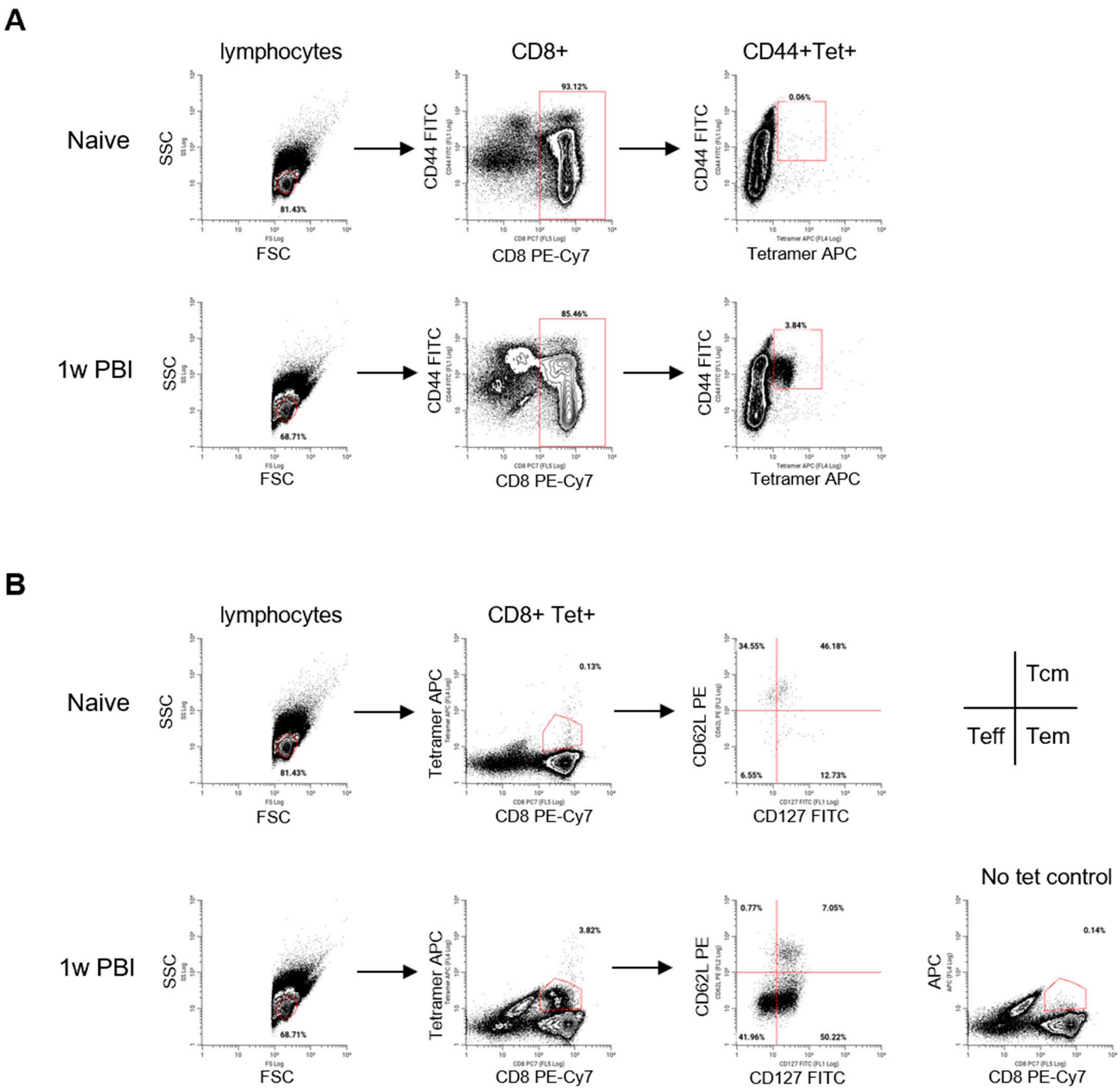

Supplemental Figure S2. Flow cytometry gating schema for CD8+Tetramer+ memory T cell phenotypic markers. Splenocytes from experimental mice were isolated and stained with antibodies to CD8, CD62L, CD127, CD44 and HPV16 E7(49-57) MHC tetramer reagent. For each sample, a no-tetramer negative control was stained. Lymphocytes were gated on FSC/SSC. Shown are representative examples of tetramer positive events in the 1 week PBI group and the naïve group. (A) Tetramer-positive cells show a phenotype of CD44+, indicating antigen-experienced T cells. (B) HPV16 E7 specific T cells identified by tetramer staining show phenotypic markers of central memory (Tcm; CD62L+CD127+)) and effector memory (Tem; CD62L-CD127+) T cells, in addition to differentiated effector T cells (Teff; CD62L-CD127-). Samples stained without tetramer were used to define positive tetramer gate position.
